# Identifying biological pathways that underlie primordial short stature using network analysis

**DOI:** 10.1530/JME-14-0029

**Published:** 2014-06

**Authors:** Dan Hanson, Adam Stevens, Philip G Murray, Graeme C M Black, Peter E Clayton

**Affiliations:** 1 Institute of Human Development, Faculty of Medical and Human Sciences, The University of Manchester Oxford Road, Manchester, M13 9WL UK; 2 Manchester Academic Health Sciences Centre (MAHSC), Central Manchester University Hospitals NHS Foundation Trust Manchester, M13 9WL UK

**Keywords:** insulin receptor, IGF, growth factors, molecular genetics

## Abstract

Mutations in *CUL7*, *OBSL1* and *CCDC8*, leading to disordered ubiquitination, cause one of the commonest primordial growth disorders, 3-M syndrome. This condition is associated with i) abnormal p53 function, ii) GH and/or IGF1 resistance, which may relate to failure to recycle signalling molecules, and iii) cellular IGF2 deficiency. However the exact molecular mechanisms that may link these abnormalities generating growth restriction remain undefined. In this study, we have used immunoprecipitation/mass spectrometry and transcriptomic studies to generate a 3-M ‘interactome’, to define key cellular pathways and biological functions associated with growth failure seen in 3-M. We identified 189 proteins which interacted with CUL7, OBSL1 and CCDC8, from which a network including 176 of these proteins was generated. To strengthen the association to 3-M syndrome, these proteins were compared with an inferred network generated from the genes that were differentially expressed in 3-M fibroblasts compared with controls. This resulted in a final 3-M network of 131 proteins, with the most significant biological pathway within the network being mRNA splicing/processing. We have shown using an exogenous insulin receptor (*INSR*) minigene system that alternative splicing of exon 11 is significantly changed in HEK293 cells with altered expression of *CUL7*, *OBSL1* and *CCDC8* and in 3-M fibroblasts. The net result is a reduction in the expression of the mitogenic INSR isoform in 3-M syndrome. From these preliminary data, we hypothesise that disordered ubiquitination could result in aberrant mRNA splicing in 3-M; however, further investigation is required to determine whether this contributes to growth failure.

## Introduction

Primordial short stature (PSS) is characterised by severe pre- and postnatal growth restriction resulting in significant short stature. There are a number of genetic syndromes that result in PSS, including the classical disorders Seckel syndrome, Meier–Gorlin syndrome and microcephalic osteodysplastic short stature types I and II (MOPD I and II) as well as the commoner normocephalic (NPSS) syndromes, 3-M and Silver–Russell syndrome (SRS) (Eggermann 2010, Clayton *et al*. 2012).

Over the past decade, genetic causes for these different PSS conditions have been successfully identified with the predicted functions of these summarised in Table 1. The importance of these pathways extends beyond growth as they also underpin other developmental processes that are associated with metabolic disease, cancer and ageing. We have extensively investigated the genetic aetiology of 3-M syndrome as a model of NPSS. Unlike many other PSS conditions, the 3-M syndrome phenotype is almost exclusively growth related with severe pre and postnatal growth restriction but no other significant system disorder (Hanson *et al*. 2011*a*). We have previously identified that mutations in three different genes *CUL7*, *OBSL1* and *CCDC8* cause 3-M syndrome (Huber *et al*. 2005, Hanson *et al*. 2009, 2011*b*, 2012). CUL7 forms the central component of an SCF E3 ubiquitin ligase (Dias *et al*. 2002) that localises to the Golgi apparatus (Litterman *et al*. 2011) and has been shown to be involved in the proteasomal degradation of IRS1 (Xu *et al*. 2008) and cyclin D1 (Okabe *et al*. 2006). Despite numerous investigations, so far additional targets of CUL7-mediated ubiquitination have remained elusive. However, it has been proposed that CUL7 may have a role in the degradation of many other proteins via its interaction with CUL1 in the formation of an ubiquitinating CUL1/CUL7 heterocomplex (Tsunematsu *et al*. 2006). OBSL1 on the other hand is a postulated cytoskeletal adaptor protein that is required for CUL7 localisation and has been implicated in the regulation of Golgi morphogenesis in neural dendrites (Litterman *et al*. 2011). Both CUL7 and CCDC8 are known interacting proteins of p53, acting as co-factors in p53-mediated apoptosis (Kim *et al*. 2007, Dai *et al*. 2011). There is little apparent similarity between the three proteins; however, the near identical phenotype of 3-M syndrome patients regardless of mutation type and the fact that OBSL1 co-immunoprecipitates with CUL7 and CCDC8 (Hanson *et al*. 2011*b*) has suggested a common biochemical pathway. In terms of the clinical and biochemical phenotype of 3-M syndrome, we have demonstrated that i) 3-M children with mutations in *CUL7* are significantly shorter than those with either *OBSL1* or *CCDC8* mutations (Hanson *et al*. 2012), ii) there is clinical evidence of GH and/or IGF1 resistance (Hanson *et al*. 2012), iii) associated with this, growth factor signalling in *ex* *vivo* 3-M fibroblast cells is disrupted (Hanson *et al*. 2012), and iv) *IGF2* expression and IGF2 secreted from 3-M fibroblasts is very low (Murray *et al*. 2013).

The mechanisms that link these observations are not defined, and therefore we have taken a ‘systems’ approach to elucidate the proteins/genes that may be implicated in the 3-M syndrome pathway. Protein–protein interactions can be mapped to create networks and in recent years larger-scale experimental workflows have been used to discover the physical interactions between different proteins allowing ever more complex interactome network models (Cho *et al*. 2004). These can range from whole organism to disease-specific interactomes (Gandhi *et al*. 2006, Lim *et al*. 2006). Known protein–protein interactions are often compiled into various databases, including Search Tool for the Retrieval of Interacting Genes/Proteins (STRING) (Franceschini *et al*. 2013) and Biological General Repository for Interaction Datasets (BioGRID) (Chatr-Aryamontri *et al*. 2013) and these along with experimental data can facilitate the mapping of biological networks.

In this study, we have used proteomic and transcriptomic approaches to identify the putative interacting partners of CUL7, OBSL1 and CCDC8 to create a 3-M syndrome interactome. These interactions have allowed us to identify key pathways and biological functions in 3-M syndrome. We have tested the impact of the most significant pathway, namely mRNA splicing, on cellular function.

## Materials and methods

### Ethics statement

Skin fibroblasts derived from 3-M syndrome patients and appropriate control individuals were used in this study. Institutional ethical approval (Central Manchester Local Research Ethics Committee 06/Q1407/21) was granted and informed written consent was obtained from all patients and control subjects. Details of samples used have been described previously (Hanson *et al*. 2012, Murray *et al*. 2013).

### Immunoprecipitation

HEK293 cells were obtained from HPA culture collection and grown under normal growth conditions in DMEM supplemented with 10% foetal bovine serum. The cells were transfected using Effectene transfection (Qiagen) reagent following the manufacturer's protocol for plasmids expressing either CUL7, V5-OBSL1 or CCDC8, which have been described elsewhere (Hanson *et al*. 2011*b*). For each of the CUL7–HEK293, V5-OBSL1–HEK293 and CCDC8–HEK293, immunoprecipitation (IP) experiments transfected HEK293 cells from six 150 mm culture dishes were lysed in ice-cold IP buffer (Pierce, Rockford, IL, USA) with protease inhibitor (Sigma) 24 h post-transfection. Protein complexes were immunoprecpitated with 5 μg of either CUL7, V5 (for OBSL1) or CCDC8 specific antibodies (Sigma; AbD Serotec, Oxford, UK; Novus Biologicals, Cambridge, UK) and collected using 100 μl of protein G Dynabeads (Invitrogen) following the manufactures recommended protocol. After washing three times in 800 μl of ice-cold IP buffer and a further two times in ice-cold PBS to remove unbound proteins, the immunocomplexes were eluted from the beads by boiling in 60 μl SDS sample buffer before separated by SDS–PAGE.

Furthermore, transfected HEK293 cells (one set of each of CUL7-HEK293, V5-OBSL1-HEK293 and CCDC8-HEK293) were immunoprecipitated in the same way, each from six 150 mm cell culture dishes except no antibody was used for the IP stage. The three samples of no antibody control IP were generated to serve as the background negative controls for mass spectrometry (MS) analysis.

The CUL7-HEK293, V5-OBSL1-HEK293 and CCDC8-HEK293 IP samples and the three background negative control IP samples were separated by SDS–PAGE. Following coomassie blue staining, gel lanes were cut into small slices (approximately ten 1 mm^3^ slices for each lane). The gel slices were dehydrated by acetonitrile (ACN), rehydrated in reduction buffer (10 mM dithiothreitol, 25 mM NH_4_HCO_3_), alkylated (55 mM iodoacetamide, 25 mM NH_4_HCO_3_) and then digested with sequencing grade trypsin (Promega). The peptides were extracted from the gel slices once with 20 mM NH_4_HCO_3_ and then twice with 5% (v/v) formic acid in 50% (v/v) ACN, samples of 20 μl concentration were ready for analysis by GeLC–MS/MS. GeLC–MS/MS analysis of the digested gel slices was carried out as described previously (Humphries *et al*. 2009).

Confirmatory IPs were carried out using transfected HEK923 cells (transfected with either CUL7, V5-OBSL1 or CCDC8 plasmids as described previously) from a single 150 mm culture dish and processed in the same manner as described earlier, using specific antibodies to CUL7, V5, CCDC8 or with no antibody as negative control IPs. Samples were separated by SDS–PAGE and immunoblotted with specific antibodies to CUL7, V5, CCDC8, HNRNPU (Santa-Cruz Biotechnology, Dallas, TX, USA), TP53 (Santa-Cruz Biotechnology), CCT2 (Cell Signaling, Danvers, MA, USA), XRCC5 (Cell Signaling) and CDK1 (Cell Signaling).

### Data analysis

#### MS data cleaning

To reduce the likelihood of false-positive results within each of the IP/MS datasets, we undertook a number of measures including removing any proteins from the datasets that only had one matching peptide sequence from MS. We conducted three separate control IPs with no antibody to remove proteins that bound non-specifically to the dynabeads used in the IP process. Proteins that were present in any of these three no antibody control IPs were subsequently removed from the CUL7, OBSL1 and CCDC8 IP/MS datasets (if present) to provide a stringent putative interacting protein list for each IP.

#### Cytoscape analysis

After removal of background interactions, to improve the stringency of the IP/MS data and because CUL7, OBSL1 and CCDC8 had previously been shown to be the components of a common biochemical complex (Litterman *et al*. 2011) suggesting they would share the majority of the same interacting partners, we identified only those proteins that were present in all three IP experiments by computing the intersection of the CUL7 IP/MS, OBSL1 IP/MS and CCDC8 IP/MS datasets for further analysis. The BioGRID database of interactions (build 3.1.103) was used to construct an ‘IP/MS network’ of known interactions between the proteins that were common to the CUL7, OBSL1 and CCDC8 IPs and this was visualised using Cytoscape (v2.8).

In tandem, we also identified gene probes that were differentially expressed between 3-M syndrome (*n*=4) and control fibroblast cells (*n*=3). RNA gene expression was assessed by Affymetrix microarray (HU-133 plus 2.0 chip) and Robust Multi-Array (RMA) analysis was used to normalise the microarray data to generate an expression level for each probe. The dataset and samples used have been described previously (Murray *et al*. 2013). For this analysis, the probes were determined to be differentially expressed if the fold change difference between 3-M and control was ±2. The resulting dataset of 913 probes (which corresponded to 683 distinct genes) was used to generate an inferred protein–protein interaction model using BioGRID, the ‘Transcriptomic network’. To improve the robustness of the IP/MS network, we took the intersection between the IP/MS and transcriptomic networks to generate a multi-omic ‘3-M interactome’. Therefore, the 3-M interactome contained only proteins that were identified to be interacting with CUL7, OBSL1 and CCDC8 and which were also shown to be associated with differential gene expression in fibroblast cells from 3-M syndrome patients compared with normal healthy controls.

We next used the Reactome database (Croft *et al*. 2011) and Webgestalt Pathway Commons (Wang *et al*. 2013) to characterise the cellular functions of the putative interacting proteins and identify over-represented biological pathways within the overall 3-M interactome. We used hypergeometric testing to determine whether the number of genes associated with each pathway identified was greater than would be expected by chance. We selected a small number of proteins from the pathways identified within the 3-M interactome, for which antibodies were available, for further IP experiments in order to confirm the interactions with CUL7, OBSL1 and CCDC8.

Key network nodes can be identified through the analysis of network properties including connectedness and centrality. We used the ModuLand cytoscape plugin to analyse the network properties of the 3-M interactome and generate clusters (or modules) represented by key network nodes. The function of these central nodes best predicts the function of the module it represents (Szalay-Beko *et al*. 2012). The central nodes are also likely to represent the key functional elements of the overall network and therefore can be used to prioritise future work.

#### Insulin receptor minigene construct

An insulin receptor (*INSR*) minigene plasmid was kindly provided as a gift by Dr Nicholas Webster, University of California San Diego. The minigene contains 110 nucleotides of exon 10, 2.2 kb of intron 10, 36 nucleotides of exon 11, 372 nucleotides of intron 11 and 103 nucleotides of exon 12. Intron 11 is a large 7.4 kb intron, but only ∼180 nucleotides were cloned at both the 5′ and 3′ ends (Talukdar *et al*. 2011). The *INSR* minigene spans a region of alternative splicing, where inclusion of exon 11 gives rise to IR-B isoform and exclusion of exon 11 to IR-A isoform.

#### Cell culture and transfections

For the *INSR* minigene assay, we used HEK293 cells and skin fibroblasts derived from 3-M syndrome patients and appropriate control individuals. Both cell types were maintained in DMEM supplemented with 10% FBS and grown at 37 °C at 5% CO_2_. HEK293 cells were transfected as previously described, with either *INSR* minigene alone or with each 3-M gene plus *INSR* minigene. While skin fibrobalsts cells (controls and cells from 3-M syndrome patients with either *CUL7*, *OBSL1* or *CCDC8* null mutations, as described previously (Hanson *et al*. 2012)) were transfected with *INSR* minigene alone.

#### RNA extraction and amplification of cDNA

The cells were harvested 24 h after transfection and total RNA was extracted using PureLink RNA mini kit (Life Technologies) following manufacturer's protocol. Contaminating genomic DNA was removed by DNase I treatment and cDNA generated following manufacturer's protocol (High capacity RNA to cDNA kit, Life Technologies). *INSR* minigene transcripts were amplified by plasmid-specific primers as described previously (Kosaki *et al*. 1998) and PCR products visualised on 4% agarose gels. Relative levels of IR-B and IR-A were assessed by gel densitometry using Image J software.

## Results

### IP/MS of CUL7, OBSL1 and CCDC8 immunocomplexes

The immunopurified protein complexes from HEK293 cells exogenously expressing either V5 tagged OBSL1, untagged CUL7 or untagged CCDC8 were analysed by in-gel liquid chromatography/tandem MS (GeLC–MS/MS) to identify the proteins binding to OBSL1, CUL7 and CCDC8. To decrease the likelihood of false-positive results, we selected only those proteins with multiple peptide matches present in the GeLC–MS/MS for inclusion in our network analysis. We identified a total of 49 proteins (Supplementary Table 1, see section on supplementary data given at the end of this article) that were present in the MS analysis of three independent negative control IPs (background IP with no antibody) and these were removed from each of the experimental datasets as false positives.

Within the resulting IP/MS datasets, we identified 618 putative CUL7-interacting proteins, 593 putative OBSL1-interacting proteins and 534 putative CCDC8-interacting proteins. There was a high degree of overlap between each of these datasets with 189 putative interacting proteins that were identified as common components in all three of the IP/MS experiments (Supplementary Table 1).

### Network analysis

To determine the likely molecular functions of the 3-M syndrome pathway and the putative interacting proteins, we used the BioGRID cytoscape plugin to create and visualise protein–protein interaction network models using the IP/MS data. Using the BioGRID database (build 103), these putative interacting proteins created a network of 176 proteins with 1031 connections between them, which we have termed the ‘IP/MS network’ (Supplementary Figure 1A, see section on supplementary data given at the end of this article).

To strengthen the validity of these interacting proteins, we simultaneously generated an interaction network using the BioGRID database derived from transcriptomic data of mutation positive 3-M syndrome patients. Using gene expression data (Murray *et al*. 2013) comparing fibroblast cells of 3-M syndrome patients (*n*=4) to age matched normal healthy control individuals (*n*=3), we identified 913 probe sets differentially expressed between 3-M syndrome patients and control samples which represented 683 distinct genes (Supplementary Table 2). The BioGRID database was used to infer an interaction network from the 683 distinct genes resulting in an overall ‘Transcriptomic network’ of 3534 proteins with 6054 connections (Supplementary Figure 1B).

**Figure 1:**
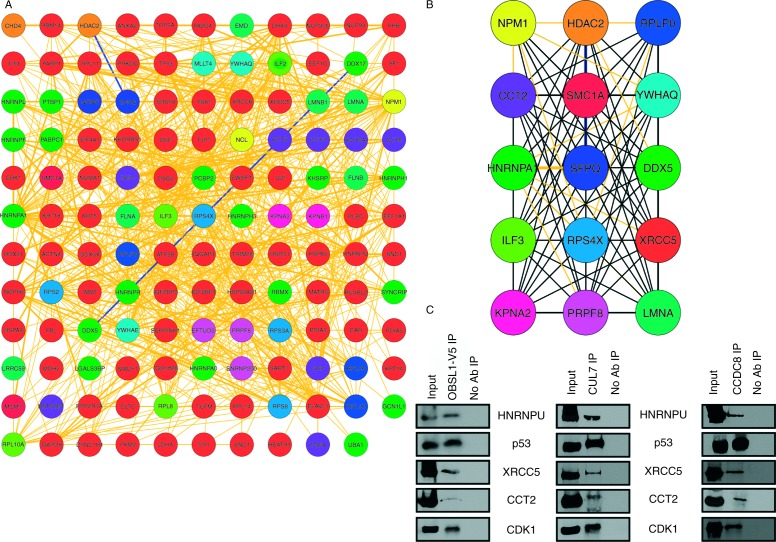
The 3-M interactome. (A) Cytoscape grid layout of the 131 proteins with 721 connections between them that form the 3-M interactome. Network was generated through identifying proteins present in both the IP/MS network and the transcriptomic network. Physical interactions are shown by orange connections and interactions which are both physical and genetic shown by blue connections. Nodes are assigned and coloured according to the central node where they most belong. (B) ModuLand network representing the key nodes within the overall network designated by degree of interactions and network centrality. (C) Immunoprecipitation of V5-OBSL1-overexpressing HEK293 cells (left panel, OBSL1-V5 IP), CUL7-overexpressing HEK293 cells (middle panel, CUL7 IP) and CCDC8 overexpressing HEK293 cells (right panel, CCDC8 IP) with western blotting to identify co-immunoprecipitated proteins to confirm the putative interactions identified by IP/MS. Protein inputs (Input) and control IPs with no antibody (no Ab IP) are shown for each panel.

We next compared the IP/MS and the trancriptomic BioGRID networks, identifying that 141 proteins were present in both networks representing a significant overlap between the two networks (hypergeometric probability, *P*=7.32×10^−61^). These 141 proteins represent the overall 3-M interactome and are proteins that were identified in the CUL7, OBSL1 and CCDC8 IP/MS datasets and within the network generated from genes that are differentially expressed in 3-M syndrome. The subsequent BioGRID network generated from the 3-M interactome contained 131 of these proteins with 721 connections (Fig. 1A).

### Pathway analysis of the 3-M interactome

We analysed the 131 proteins from the BioGRID-derived network to identify the cellular pathways that are associated with the 3-M interactome. This pathway analysis showed significant over-representation of mRNA splicing/processing, metabolism of proteins, cell cycle, apoptosis and DNA repair pathways (Tables 2 and 3). In addition Webgestalt analysis also identified an over-representation of a number of signalling pathways most notably the Insulin, IGF1, VEGF and mTOR pathways (Table 3). At an individual protein level, we identified that ten of the 20 known major heterogeneous ribonucleoprotein (HNRNP) complex proteins (Chaudhury *et al*. 2010) along with other RNA-binding proteins and ribosomal subunit proteins in particular were amongst the most abundant within the combined 3-M interactome. The network properties including node (protein) centrality and connectivity were used to determine community centrality of each node within the 3-M interactome. This was assessed by the ModuLand method to identify the nodes which best represent the function of the overall network and revealed 15 key 3-M interactome modules (or node centres) (Fig. 1B).

### Additional IPs to confirm interactions

We next performed additional IPs in HEK293 cells overexpressing CUL7, V5-OBSL1 and CCDC8 using specific antibodies to either CUL7, V5 or CCDC8. In each of the CUL7, V5-OBSL1 and CCDC8 IPs we were able to recover proteins within a number of the key pathways associated with the network as confirmation of their association within the 3-M interactome which were not present in the ‘no antibody control’ IPs. This includes two central nodes identified by ModuLand, XRCC5 and CCT2. We confirmed interactions with proteins in a number of pathways, including mRNA splicing/processing (HNRNPU), metabolism of proteins and protein folding (CCT2), double-strand repair, Non-homologous end-joining (XRCC5) and cell cycle (TP53 and CDK1) (Fig. 1C).

### CUL7, OBSL1 and CCDC8 modulate the alternative splicing of the INSR

**Figure 2:**
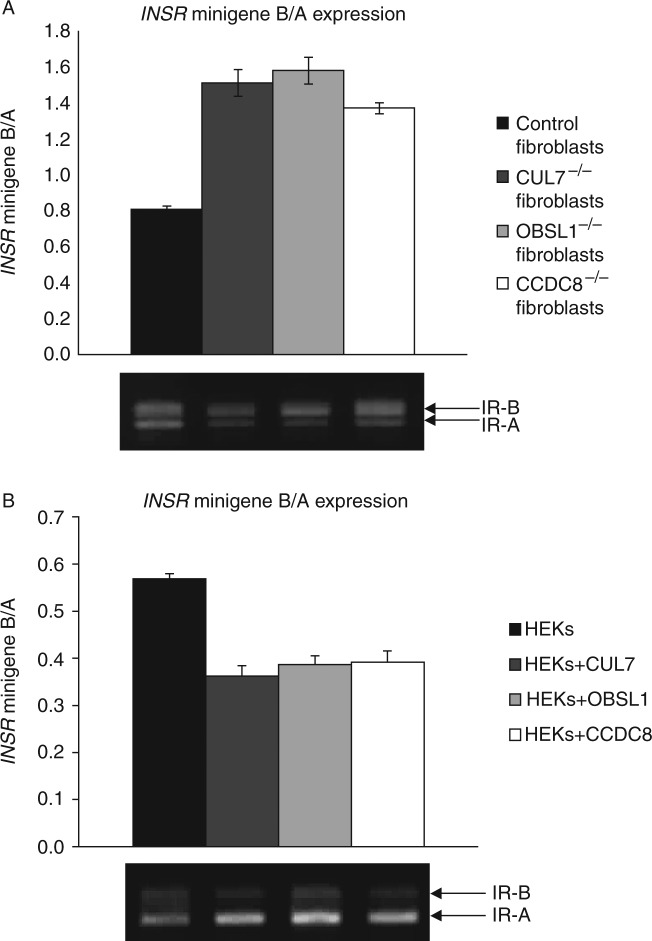
INSR minigene assay. (A) Quantification of alternative splicing of *INSR* minigene in fibroblast cells. Control cells (*n*=3) and fibroblasts from 3-M syndrome patients, CUL7^−/−^, OBSL1^−/−^ and CCDC8^−/−^, were transfected with an *INSR* minigene construct and relative levels of INSR were measured by RT-PCR analysis. Graph indicates the relative expression of IR-B/IR-A as a mean for *n*=10 transfection experiments for each cell type, a representative gel is shown below the graph. Error bars represent s.e.m. (B) Quantification of alternative splicing of *INSR* minigene in HEK293 cells. HEK293 cells were transfected with *INSR* minigene construct only (labelled HEKs, *n*=8 transfection experiments) or with minigene and a CUL7 expression vector (HEKs+CUL7, *n*=5 transfection experiments), with minigene and a OBSL1 expression vector (HEKs+OBSL1, *n*=5 transfection experiments) and with minigene and a CCDC8 expression vector (HEKs+CCDC8, *n*=5 transfection experiments). Graph indicates the mean relative expression of IR-B/IR-A for each combination of transfections as indicated, a representative gel is shown below the graph. Error bars represent s.e.m.

RNA splicing is the most significantly associated cellular pathway within the 3-M interactome and HNRNP proteins are amongst the most common components of this pathway. We have confirmed the interaction of HNRNPU with all three 3-M proteins and also identified that HNRNPA1 and HNRNPF are in the 3-M interactome. Talukdar *et al*. (2011) have recently demonstrated that HNRNP F, H1 and U bind to the splicing motif of intron 10 of *INSR* and where HNRNPA1 promotes exon 11 exclusion and HNRNPF promotes exon 11 inclusion. The alternative splicing of *INSR* gives rise to two different protein isoforms IR-A (− exon 11) and IR-B (+ exon 11) (Belfiore *et al*. 2009). To determine if CUL7, OBSL1 and CCDC8, through their interaction with HNRNPs and other members of the splicing machinery, also to regulate alternative splicing events, we have used an *INSR* minigene system to determine the effect of the 3-M proteins on the inclusion/exclusion of exon 11 of *INSR*. In fibroblast cells from normal control patients and those derived from 3-M syndrome patients we show that loss of CUL7, OBSL1 or CCDC8 leads to a reduction in IR-A isoform and therefore an increase in the ratio of IR-B to IR-A expression (Fig. 2A). Conversely overexpression of CUL7, OBSL1 or CCDC8 in HEK293 cells results in an increase in IR-A expression and subsequent decrease in IR-B to IR-A ratio (Fig. 2B).

## Discussion

In this study, we have been able to combine experimental IP/MS and transcriptomic data from 3-M syndrome patients to generate a disease interactome. We have associated molecular pathways with this interactome to identify biological processes that underlie this PSS condition. Some of the proteins identified in this study, which form the 3-M interactome, are likely to be ideal candidate short stature genes that may be defective in undiagnosed 3-M syndrome or in similar PSS disorders. The association of molecular pathways with the 3-M syndrome proteins has given us further insights into the molecular mechanisms of growth restriction seen in this condition and potentially other short stature disorders.

There are potential limitations of using an IP/MS approach to identify the interacting partners of a particular protein; this includes the possibility of identifying both direct and indirect interactions. Future studies, for example, utilising Forster resonance energy transfer (FRET) experiments between the 3-M proteins and a number of the key interacting partners could determine whether these are direct interactions and therefore directly associated with the 3-M pathway. Nevertheless, it is clear that there is a strong association of RNA processing, ribosome and cell cycle pathways within the CUL7, OBSL1 and CCDC8 networks. In particular, in each of the IP/MS datasets there was a high proportion of RNA binding/processing proteins with a highly significant probability of enrichment in pathways associated with either RNA processing or splicing (Supplementary Table 1) and therefore likely that at least some of these would be direct interactions. The association of RNA binding proteins was also supported by additional IP of HNRNPU with all three 3-M proteins (Fig. 1C).

The possibility of false-positive interactions is often regarded as a weakness with MS-derived data. We used a stringent analysis protocol in which only proteins that were present in all three experimental IPs but not in any of the three negative-control IPs were identified as potential interacting proteins. To further increase confidence in our data, we used a multi-omic approach using transcriptomic data from 3-M syndrome patients' fibroblast cells alongside the IP/MS data. The common proteins within these datasets defined the overall 3-M syndrome interactome. As a measure of the robustness of the analysis we applied to the IP/MS data, there was a high degree of overlap between the IP/MS and transcriptomic data with 141 of the 189 proteins in the IP/MS data also present in the transcriptomic network.

Our data is in alignment with recent studies on the function of the different 3-M proteins; Litterman *et al*. (2011) recently demonstrated that OBSL1 is a major component of the CUL7 SCF complex which also includes an F-box specificity factor, FBXW8. These IP studies identified that five members of the T-complex protein 1 (TCP1) chaperonin complex (CCT2, CCT3, CCT6A, CCT6B and CCT7) are putative interacting partners of FBXW8. Supporting this observation, we also found four members of this protein family (TCP1, CCT2, CCT3 and CCT6A) were present in the 3-M interactome and predict they may act as adaptor proteins within the CUL7 SCF complex. IP experiments from lysates of HEK293 cells overexpressing CUL7, V5-OBSL1 and CCDC8 confirmed the interaction between CCT2 and the 3-M proteins and CCT2 was also one of the key network nodes within the 3-M interactome.

P53 is a major tumour suppressor gene that is vital for maintaining normal cell growth and in particular is central to the stress response of cells (Steele *et al*. 1998). Numerous studies have identified that CUL7 interacts with p53 and that the CUL7 SCF complex is able to monoubiquitinate p53; however, it is unlikely to be a true proteasomal degradation substrate (Andrews *et al*. 2006, Kasper *et al*. 2006, Kaustov *et al*. 2007). Knockdown of *CUL7* increases p53-mediated inhibition of cell cycle progression, while *CUL7* overexpression represses p53 induction after DNA damage suggesting *CUL7* is an antiapoptotic oncogene (Jung *et al*. 2007, Kim *et al*. 2007). Acetylation of p53 by KAT5 (also known as Tip60) is thought to play a role in the activation of p53 in stress response and induces p53-mediated apoptosis. Recently CCDC8 was shown to interact with both p53 and KAT5 and is required for activation of BBC3 (also known as PUMA) during p53-mediated apoptotic response (Dai *et al*. 2011). Our IP studies support the interaction between CUL7 and p53 along with the interaction between CCDC8 and p53 while also implying that OBSL1 associates with p53 as part of this complex.

In some MPSS disorders, mutations in genes associated with DNA damage and cell cycle have been identified. This includes mutations in the DNA damage response kinase *ATR* as a cause of Seckel syndrome and *PCNT* mutations, which have been identified in MOPDII. Cell lines derived from patients with *PCNT* mutations have been shown to have disrupted signalling of ATR-dependent DNA damage response. CDK1 is a key regulator of the ATR signalling pathway required for G2/M transition. It has been shown previously that mutations in *ATR*, *ATRIP* and *CEP152* associated with PSS results in loss of function of these genes which impairs the activity of the ATR signalling pathway and therefore alters the G2/M checkpoint (Klingseisen & Jackson 2011). Our 3-M interactome identified that a number of cell cycle and DNA damage response proteins are associated with 3-M proteins, resulting in significant over-representation of these pathways (Tables 2 and 3). CDK1 was also confirmed as an interacting partner of the 3-M proteins. Consistent with the role of CUL7, OBSL1 and CCDC8 as growth-promoting genes, and their association with cell cycle proteins, we have previously shown that fibroblast cells from 3-M syndrome patients with null mutations in the 3-M genes have a significantly reduced level of cell proliferation compared with normal control fibroblast cells (Murray *et al*. 2013). Our analysis of the 3-M interactome identified that the DNA damage response protein XRCC5 was also one of key central network nodes (Fig. 1B). The role the 3-M proteins have on XRCC5 function and DNA damage response is not characterised; however, there is evidence that elevated expression of *CUL7* is associated with cancer progression and poor survival (Kim *et al*. 2007).

The most significantly associated pathways in the 3-M interactome are those that are involved in the regulation of mRNA splicing. Mutations in splicing proteins have previously been associated with primordial dwarfism for which mutations in *RNU4ATAC* cause MOPDI (Nagy *et al*. 2012). We have shown that overexpression of CUL7, OBSL1 and CCDC8 results in an increase in IR-A expression in HEK293 cells as a result of increased levels of exon 11 exclusion in a minigene system. We also found that knockout of *CUL7*, *OBSL1* or *CCDC8* in 3-M patient fibroblast cell models show a reduction in IR-A expression of the *INSR* minigene.

IR-A predominantly mediates the mitogenic activity of insulin, whereas IR-B predominantly mediates the metabolic effects (Belfiore *et al*. 2009). Furthermore, IR-A is associated with increased proliferative rates, and elevated IR-A is found in both foetal and cancer tissues (Belfiore *et al*. 2009). The Insulin and IGF1 pathways are amongst the pathways most commonly associated with the 3-M interactome (Table 3) and we have previously demonstrated that 3-M syndrome patients show defective phosphorylation of AKT and MAPK upon growth factor stimulation and clinically there is a suggestion that 3-M patients have a degree of GH and/or IGF1 resistance (Hanson *et al*. 2012). IRS-1 is an important adaptor molecule downstream of the insulin, IGF1, and GH receptors and it has also been shown to be a target of the CUL7 SCF complex resulting in the dysfunction of AKT and MAPK signalling cascades (Xu *et al*. 2008).

Although preliminary these studies suggests that the 3-M proteins themselves could be involved in the modulation of alternative splicing of *INSR*. However, in light of the already known abnormalities within the IGF system, it remains to be established whether the proposed modulation of *INSR* splicing has any direct impact on the growth failure seen in 3-M syndrome patients. Future studies could look to determine if the association of 3-M proteins with components of the major splicing pathways has a more global effect on alternative splicing events, in particular on other pathways identified in the 3-M interactome, and whether this may contribute to the pathology.

3-M syndrome patients are typically born small for gestational age as a result of foetal growth restriction. Our previously published transcriptomic data from 3-M syndrome patients with null mutations in either *CUL7*, *OBSL1* or *CCDC8* revealed that *IGF2* expression is significantly reduced (Murray *et al*. 2013). The 3-M interactome data suggest that this could be facilitated by the direct interaction that we have identified with both IGF2BP1 and IGF2BP3, which are known to interact with the *IGF2* 5′UTR. SRS is clinically similar to 3-M syndrome and has been associated with epigenetic alterations of the IGF2/H19 locus resulting in the loss of *IGF2* expression (Eggermann 2010). Our association of the 3-M syndrome proteins with this pathway may suggest that defects in the IGF system underlie these phenotypically similar NPSS conditions.

## Conclusion

**Figure 3:**
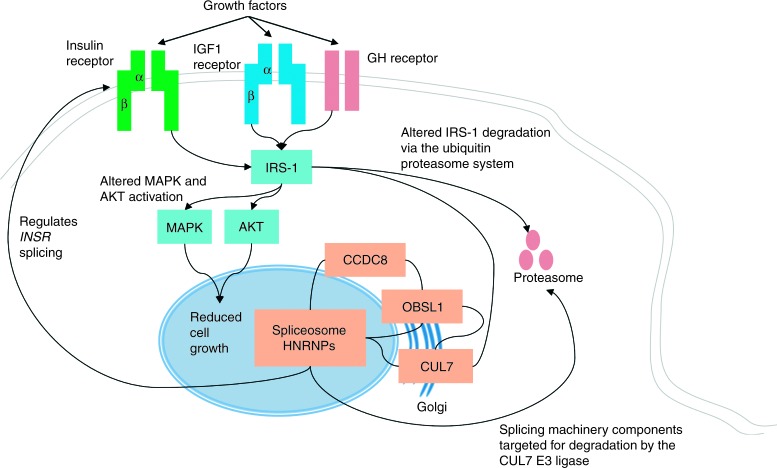
The CUL7-OBSL1-CCDC8 pathway and its predicted role in cell growth. OBSL1 interacts with both CUL7 and CCDC8 (solid connections shows protein–protein interactions) all three associate with the mRNA splicing machinery with particularly high abundance of HNRNPs in the 3-M interactome. Alternative splicing of the Insulin receptor (*INSR*) is modulated by CUL7, OBSL1 and CCDC8, IRS-1 is also a target of the CUL7 E3 ubiqutin ligase and this impacts on downstream signalling upon growth factor stimulation leading to dysfunction in MAPK and AKT activation. This subsequently results in a reduction of cell proliferation in cells derived from 3-M syndrome patients.

Our multi-omic approach alongside previous studies has identified a strong association of mRNA splicing, ubiquitination and the IGF pathway with the function of the CUL7/OBSL1/CCDC8 complex. We have also identified an association with cell cycle and DNA damage response pathways which are also found to be defective in numerous other PSS orders suggesting that their dysfunction is vital for postnatal growth. We postulate that the interactions of the 3-M proteins we have identified may link the disruption of CUL7 SCF substrate ubiquitination and their subsequent accumulation in 3-M syndrome to alteration of major splicing events. This may in turn lead to dysfunction of growth factor signalling, resulting in growth restriction via altered cell cycle progression and DNA damage response (Fig. 3).

## Supplementary data

This is linked to the online version of the paper at http://dx.doi.org/10.1530/JME-14-0029.

Supplementary Data

Supplementary Data

## Figures and Tables

**Table 1:** Summary of the genetic causes of primordial short stature disorders

**Primordial short stature condition**	**Genetic causes**	**Postulated function**
Normocephalic		
3-M syndrome	*CUL7*, *OBSL1*, *CCDC8*	Cullin E3 ubiquitin ligase which targets IRS1 and cyclin D1 for proteasomal degradation
Silver–Russell syndrome	11p15 *H19/IGF2* hypomethylation, maternal UPD7	Imprinting defects which affect expression of the foetal growth factor IGF2
Microcephalic		
Seckel syndrome	*ATR*, *ATRIP*, *CENPJ*, *CEP152*	DNA damage response and centriole biogenesis
Meier–Gorlin syndrome	*ORC1*, *ORC4*, *ORC6*, *CDT1*, *CDC6*	DNA replication complex
MOPDI	*RNU4ATAC*	Minor spliceosome
MOPDII	*PCNT*	Centrosome and DNA damage response

**Table 2:** Reactome analysis of the 3-M interactome

**Un-adjusted probability of seeing N or more genes in this Event by chance**	**Number of genes in your query which map to this event**	**Total number of genes involved in this event**	**Name of this event**	**Submitted identifiers mapping to this event**
1.67×10^−13^	17	112	mRNA splicing	SNRNP200, PTBP1, YBX1, SMC1A, HNRNPA0, HNRNPF, HNRNPH1, PRPF8, EFTUD2, DHX9, PCBP2, SRSF9, HNRNPA1, HNRNPL, HNRNPU, RBMX, HNRNPR
4.37×10^−12^	17	136	mRNA processing	SNRNP200, PTBP1, YBX1, SMC1A, HNRNPA0, HNRNPF, HNRNPH1, PRPF8, EFTUD2, DHX9, PCBP2, SRSF9, HNRNPA1, HNRNPL, HNRNPU, RBMX, HNRNPR
3.64×10^−10^	40	1031	Gene expression	SNRNP200, PTBP1, IGF2BP3, YBX1, RPS3A, ELAVL1, RPLP0, HNRNPA0, RPL18, HNRNPF, EEF1G, EEF1A1, IGF2BP1, RPL14, RPS4X, RPS2, PCBP2, RPS8, HNRNPA1, RPL10A, PABPC1, HNRNPR, EIF4A1, SF1, SMC1A, HNRNPH1, RPL11, PRPF8, RPL7A, EFTUD2, PARP1, KHSRP, PPP2R1A, DHX9, SRSF9, RPL8, HNRNPU, HNRNPL, RBMX, TRIM28
6.24×10^−10^	29	574	Metabolism of proteins	EIF4A1, HSPD1, RPS3A, LMNA, CCT6A, CCT3, RPLP0, RPL18, PDIA3, RPL11, EEF1G, EEF1A1, HSP90B1, CCT2, RPL7A, HSPA5, PDIA6, CCT8, TCP1, RPL14, RPS4X, RPS2, RPL8, RPS8, HSPA9, ATP5B, RPL10A, NOP56, PABPC1
3.21×10^−9^	13	109	3′-UTR-mediated translational regulation	EIF4A1, RPL7A, RPS3A, RPL14, RPS4X, RPS2, RPL8, RPS8, RPLP0, RPL18, RPL10A, PABPC1, RPL11
5.37×10^−5^	3	6	Nonhomologous end joining (NHEJ)	XRCC5, PRKDC, XRCC6
5.90×10^−5^	10	154	Apoptosis	LMNB1, CAD, LMNA, TJP1, YWHAE, DSG2, YWHAQ, DSP, KPNB1, PLEC
9.36×10^−5^	6	53	Protein folding	CCT2, CCT8, NOP56, TCP1, CCT6A, CCT3
0.000684505	8	137	Cell–cell communication	FLNA, ACTN4, MLLT4, JUP, KRT14, IQGAP1, KRT5, PLEC
0.001024479	16	478	Cell cycle	LMNB1, DYNC1H1, LMNA, CDK1, SMC1A, TOP2A, TP53, EMD, TPR, PPP2R1A, NUP93, MCM7, YWHAE, NUMA1, NUP205, NPM1
0.001530044	14	403	Cell cycle, mitotic	LMNB1, EMD, TPR, DYNC1H1, PPP2R1A, LMNA, CDK1, YWHAE, SMC1A, MCM7, NUP93, NUMA1, TOP2A, NUP205
0.003057906	3	21	Double-strand break repair	XRCC5, PRKDC, XRCC6
0.004274334	10	266	Mitotic M-M/G1 phases	LMNB1, EMD, TPR, PPP2R1A, LMNA, CDK1, SMC1A, MCM7, NUP93, NUP205
0.016112501	21	915	Disease	RPS3A, CDK1, RPLP0, RPL18, RPL11, KPNB1, RPL7A, TPR, PPP2R1A, RPL14, RPS2, RPS4X, NUP93, RPL8, XRCC5, RPS8, HDAC2, RPL10A, NUP205, XRCC6, NPM1

**Table 3:** WebGestalt analysis of the 3-M interactome

**Adjusted probability of seeing N or more genes in this event by chance**	**Number of genes in your query which map to this event**	**Total number of genes involved in this event**	**Name of this event**	**Submitted identifiers mapping to this event**
3.39×10^−41^	36	379	Gene expression	RPS8, HNRNPA1, PRPF8, RPS2, DHX9, HNRNPA0, HNRNPH1, EEF1G, EEF1A1, NUP205, RPL11, RPL18, EFTUD2, YBX1, HNRNPL, HNRNPU, PABPC1, PCBP2, SNRNP200, PTBP1, HNRNPF, RPL8, EIF4A1, RPL10A, TRIM28, RPLP0, RPS3A, RPS4X, RPL7A, TPR, HNRNPR, RBMX, NUP93, RPL14, SMC1A, SRSF9
2.99×10^−25^	20	157	mRNA processing	HNRNPA1, PRPF8, SNRNP200, PTBP1, DHX9, HNRNPF, HNRNPA0, HNRNPH1, NUP205, TPR, HNRNPR, RBMX, EFTUD2, NUP93, SMC1A, HNRNPL, YBX1, HNRNPU, SRSF9, PCBP2
3.11×10^−25^	38	1304	VEGF and VEGFR signalling network	HNRNPA1, HSP90B1, JUP, COPA, IQGAP1, TP53, NPM1, TJP1, YWHAE, RPL11, CAD, PA2G4, PPP2R1A, IGF2BP1, HSPD1, ENO1, KPNB1, KRT5, GAPDH, PRKDC, HSP90AB1, KRT14, LDHA, YWHAQ, EIF4A1, NCL, DSP, CDK1, TRIM28, XRCC5, MLLT4, CLTC, XRCC6, ACTN4, DDX5, KPNA2, RUVBL2, HDAC2
6.45×10^−25^	37	1288	Insulin pathway	HNRNPA1, HSP90B1, JUP, COPA, IQGAP1, TP53, NPM1, TJP1, YWHAE, RPL11, CAD, PA2G4, PPP2R1A, IGF2BP1, HSPD1, ENO1, KPNB1, KRT5, GAPDH, PRKDC, KRT14, LDHA, YWHAQ, EIF4A1, NCL, DSP, CDK1, TRIM28, XRCC5, MLLT4, CLTC, XRCC6, ACTN4, DDX5, KPNA2, RUVBL2, HDAC2
6.45×10^−25^	37	1288	mTOR signalling pathway	HNRNPA1, HSP90B1, JUP, COPA, IQGAP1, TP53, NPM1, TJP1, YWHAE, RPL11, CAD, PA2G4, PPP2R1A, IGF2BP1, HSPD1, ENO1, KPNB1, KRT5, GAPDH, PRKDC, KRT14, LDHA, YWHAQ, EIF4A1, NCL, DSP, CDK1, TRIM28, XRCC5, MLLT4, CLTC, XRCC6, ACTN4, DDX5, KPNA2, RUVBL2, HDAC2
6.45×10^−25^	37	1291	IGF1 pathway	HNRNPA1, HSP90B1, JUP, COPA, IQGAP1, TP53, NPM1, TJP1, YWHAE, RPL11, CAD, PA2G4, PPP2R1A, IGF2BP1, HSPD1, ENO1, KPNB1, KRT5, GAPDH, PRKDC, KRT14, LDHA, YWHAQ, EIF4A1, NCL, DSP, CDK1, TRIM28, XRCC5, MLLT4, CLTC, XRCC6, ACTN4, DDX5, KPNA2, RUVBL2, HDAC2
4.81×10^−24^	17	107	mRNA splicing	HNRNPA1, PRPF8, SNRNP200, PTPB1, DHX9, HNRNPF, HNRNPA0, HNRNPH1, HNRNPR, RBMX, EFTUD2, SMC1A, HNRNPL, YBX1, HNRNPU, SRSF9, PCBP2
3.05×10^−23^	21	261	Metabolism of proteins	RPS8, CCT2, RPS2, RPL8, CCT3, PDIA3, EIF4A1, RPL10A, EEF1G, RPLP0, CCT8, EEF1A1, RPS3A, RPL11, RPL7A, RPS4X, RPL18, TCP1, RPL14, CCT6A, PABPC1
3.59×10^−17^	13	103	3′-UTR-mediated translational regulation	RPL7A, RPS8, RPS4X, RPS2, RPL18, RPL8, RPL14, EIF4A1, RPL10A, RPLP0, PABPC1, RPS3A, RPL11
8.37×10^−11^	11	200	Wnt signalling network	HNRNPA1, PA2G4, PRKDC, XRCC6, YWHAQ, RUVBL2, FLNA, IGF2BP1, YWHAE, HDAC2, XRCC5
2.53×10^−9^	5	16	Chaperonin-mediated protein folding	CCT3, CCT6A, CCT2, TCP1, CCT8
7.28×10^−8^	8	158	Apoptosis	TJP1, PLEC, LMNB1, DSP, KPNB1, DSG2, LMNA, TP53
6.35×10^−7^	3	5	Nonhomologous end joining (NHEJ)	PRKDC, XRCC6, XRCC5
3.62×10^−6^	6	117	Cell–cell communication	ACTN4, MLLT4, PLEC, FLNA, JUP, IQGAP1
1.26×10^−5^	8	318	Cell cycle, mitotic	DYNC1H1, TOP2A, NUMA1, PPP2R1A, SMC1A, YWHAE, MCM7, CDK1
2.65×10^−5^	5	99	Mitotic G2-G2/M phases	DYNC1H1, NUMA1, YWHAE, PPP2R1A, CDK1
0.0001	3	27	Double-strand break repair	PRKDC, XRCC6, XRCC5
