# Supplementary material for: Identifying biological pathways that underlie primordial short stature using network analysis
Source: J Mol Endocrinol. 2014 Apr 7;52(3):333–44. doi: 10.1530/JME-14-0029 (PMC4045235; doi:10.1530/JME-14-0029)
Supplement: Supplementary Data [file supp_JME-14-0029_Supplementary_figure_1.pdf]

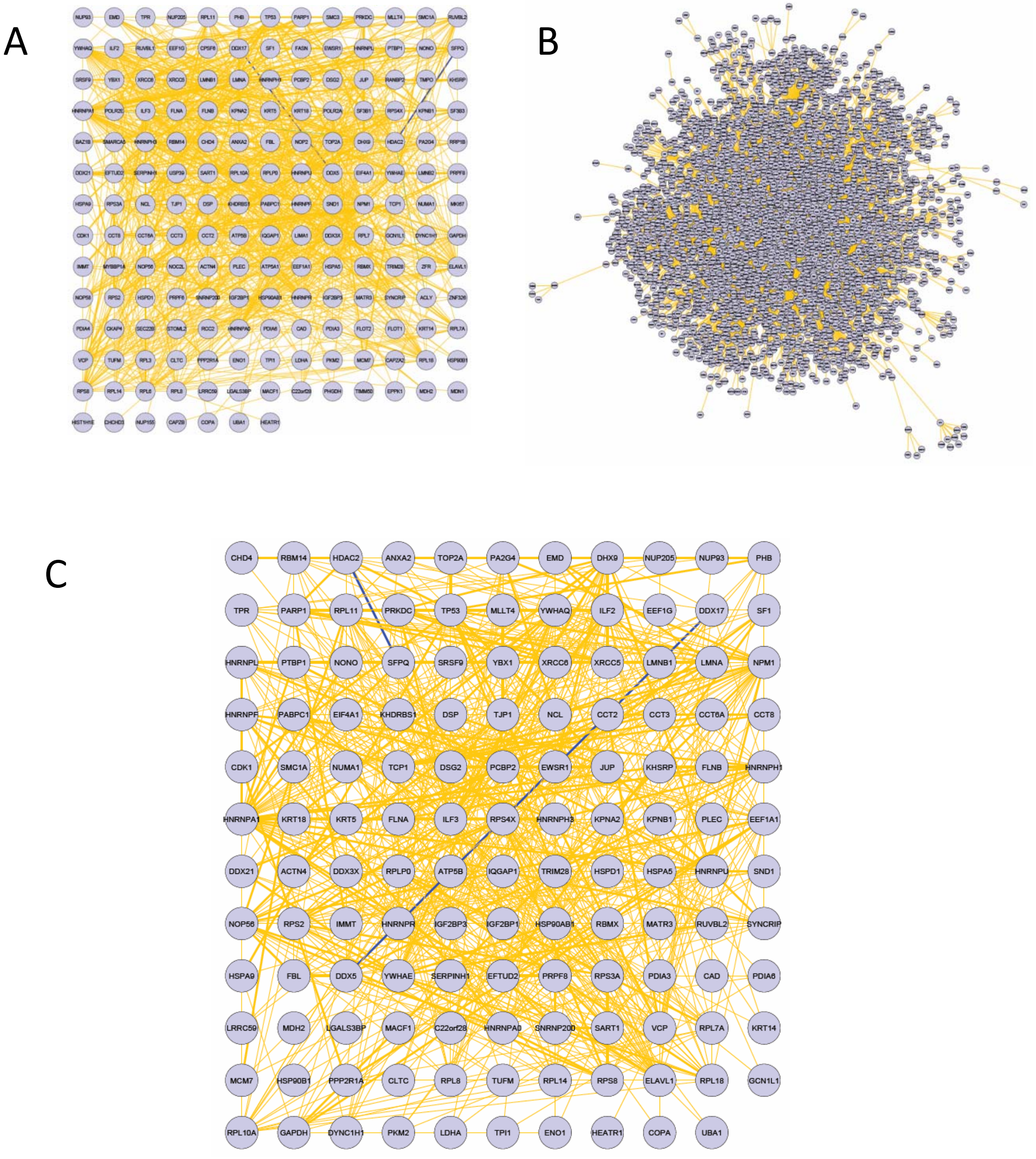

Supplementary Figure: BioGRID (build 3.1.103) derived networks. A. IP/MS interactome from overlap of individual immunoprecipitation experiments, 176 proteins with 1031 connections. B. Transcriptomic interactome, 3534 proteins with 6054 connections. C. Intersection of A and B representing the overall 3-M interactome, 131 proteins with 721 connections. Physical interactions in orange, physical and genetic interactions in blue.
