# Supplementary material for: Identifying biological pathways that underlie primordial short stature using network analysis
Source: J Mol Endocrinol. 2014 Apr 7;52(3):333–44. doi: 10.1530/JME-14-0029 (PMC4045235; doi:10.1530/JME-14-0029)
Supplement: Supplementary Data [file supp_52_3_333_v2_index.html]

Identifying biological pathways that underlie primordial short stature using network analysis — Identifying biological pathways that underlie PSS — Supplementary Data 

# Identifying biological pathways that underlie primordial short stature using network analysis

## Supplementary Data

**Files in this Data Supplement:**

- Supplementary Figure 1 - (PDF 564 KB)
- Supplementary Table 1 - (XLS 69 KB)
- Supplementary Table 2 - (XLS 143 KB)
